# Specific decellularized extracellular matrix promotes the plasticity of human ocular surface epithelial cells

**DOI:** 10.3389/fmed.2022.974212

**Published:** 2022-11-15

**Authors:** Tiago Ramos, Mohit Parekh, Paula Meleady, Finbarr O’Sullivan, Rosalind M. K. Stewart, Stephen B. Kaye, Kevin Hamill, Sajjad Ahmad

**Affiliations:** ^1^Department of Eye and Vision Science, University of Liverpool, Liverpool, United Kingdom; ^2^Faculty of Brain Sciences, Institute of Ophthalmology, University College London, London, United Kingdom; ^3^Primary Department, National Institute for Cellular Biotechnology, Dublin City University, Dublin, Ireland; ^4^St Paul’s Eye Unit, Royal Liverpool University Hospital, Liverpool, United Kingdom; ^5^Department of Ophthalmology, Aberdeen Royal Infirmary, Aberdeen, United Kingdom; ^6^External Eye Disease Service, Moorfields Eye Hospital, London, United Kingdom

**Keywords:** cell differentiation, transdifferentiation, cellular reprograming, epithelial cell, extracellular matrix, laminin, ocular surface

## Abstract

The ocular surface is composed of two phenotypically and functionally different epithelial cell types: corneal and the conjunctival epithelium. Upon injury or disease, ocular surface homeostasis is impaired resulting in migration of conjunctival epithelium on to the corneal surface. This can lead to incomplete transdifferentiation toward corneal epithelial-like cells in response to corneal basement membrane cues. We show that corneal extracellular matrix (ECM) proteins induce conjunctival epithelial cells to express corneal associated markers losing their conjunctival associated phenotype at both, mRNA and protein level. Corneal epithelial cells behave the same in the presence of conjunctival ECM proteins, expressing markers associated with conjunctival epithelium. This process of differentiation is accompanied by an intermediate step of cell de-differentiation as an up-regulation in the expression of epithelial stem cell markers is observed. In addition, analysis of ECM proteins by laminin screening assays showed that epithelial cell response is laminin-type dependent, and cells cultured on laminin-511 showed lower levels of lineage commitment. The phosphorylation and proteolysis levels of proteins mainly involved in cell growth and differentiation showed lower modifications in cells with lower lineage commitment. These observations showed that the ECM proteins may serve as tools to induce cell differentiation, which may have potential applications for the treatment of ocular surface injuries.

## Introduction

**FIGURE 1:**
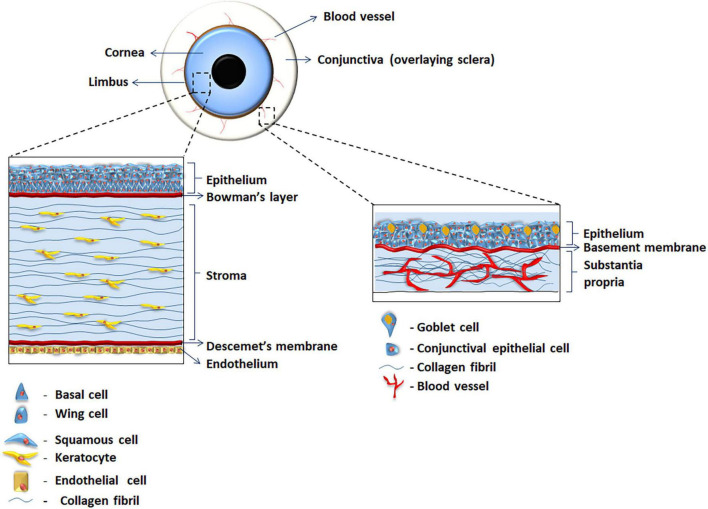
Illustration of the human ocular surface showing the anatomy of human cornea and conjunctiva.

The anterior surface of the eye is composed of two phenotypical and functionally different structures: the cornea and the conjunctiva. The cornea is a specialized transparent avascular structure covered by the corneal epithelium that rests on a collagenous layer (Bowman’s membrane). The anterior sclera that encircles the cornea is covered by the conjunctiva with an epithelium that extends to cover the inner surface of the eyelids. Physically separating the cornea and the conjunctiva is a narrow band of tissue known as the limbus which encircles the cornea and also harbors the stem cells (SCs) for the corneal epithelium, known as limbal stem cells (LSC) (1, 2) (Figure 1). The conjunctival stem cells are conversely scattered throughout the conjunctival tissue with highest concentrations in the medial bulbar and inferior forniceal regions (3). Differences in the microenvironments of the corneal and limbal epithelium are known to exist with regards to basement membrane (BM) composition (4–11). The loss or dysfunction of LSCs results in the imbalance of corneal-conjunctival homeostasis that frequently leads to a process of re-epithelization of the corneal surface by the conjunctival epithelium, known as conjunctivalization (12, 13). This is accompanied by chronic inflammation, corneal scarring and vascularization (14), resulting in vision loss and severe discomfort (15). Mann and Pullinger (16) and later Friedenwald et al. (17) demonstrated that conjunctival epithelial cells (ECs) that migrate on to the cornea acquire an incomplete corneal-like phenotype being trapped in an incomplete metaplastic transition (later designated conjunctival transdifferentiation) between the corneal and the conjunctival type (18). Other studies have shown that the migrating conjunctival cells differ from the normal corneal epithelium in glycogen metabolism and protein profiling (19–22). Kurpakus et al. suggested that conjunctival transdifferentiation is the result of environmental modulation, with the corneal BM playing a key role in such process (23). Corneal and conjunctival epithelial cells (ECs) belong to two phenotypically different lineages (24), they express different keratins (KRTs) and have differing cell-cell and cell-matrix adhesions. KRTs are intermediate filaments (type I and type II) that aid to form the cytoskeleton of ECs providing them with structural integrity. KRT3 and KRT12 are the most widely accepted ones for corneal ECs (25–27), whereas KRT7 and KRT13 are accepted for the conjunctival ECs (25, 28, 29). Functionally, as compared to corneal epithelium, that is clearer and more tightly adherent, the conjunctival epithelium is hazier, has fewer tight junctions and is less adherent to its BM (9, 30). We therefore aim to show that ECM cues play a crucial role in modulating cell differentiation. The conjunctival and corneal microenvironments were recapitulated *in vitro* to promote conjunctival differentiation toward corneal epithelial-like lineages, and vice-versa.

## Materials and methods

### Ethical statement

Ethical approval was obtained, and the study was performed in accordance to the standards of the 1964 Declaration of Helsinki. Informed consent was obtained from donors’ next-of-kin prior to their inclusion in the study. Donor median age was 84 years old, and post-mortem eye retrieval was 10 to 22 h. Cadaveric limbal tissue (4 donors, 6 eyes), remaining after removal of the central cornea for transplantation purposes and composed of peripheral cornea and limbus, and cadaveric conjunctival tissue (bulbar and forniceal regions from 6 donors, 10 eyes) was obtained from eye bank sources.

### Cell culture

The Human conjunctival epithelial cell line (HCjE-Gi) (conjunctival) cell line was kindly gifted by Ilene Gipson (Schepens Eye Research Institute, USA). These cells were cultured on keratinocyte serum free media (KSFM) without CaCl_2_ (Gibco™ ThermoFisher Scientific, cat. Number 37010-022), supplemented with 0.2% (V/V) Bovine Pituitary Extract (BPE), 0.2ng/mL of epithelial growth factor (EGF) (all supplied with the medium), 1% (V/V) penicillin/streptomycin (P/S, Sigma-Aldrich, cat. Number P0781), and 0.4mM of CaCl_2_ (Sigma-Aldrich, cat. Number 21114). The Human corneal epithelial cell line (hTCEpi) (corneal) cell line was kindly gifted by Professor James Jester (University of Southern California, USA). These cells were cultured in KSFM (Gibco™ ThermoFisher Scientific, cat. Number 17005-042), supplemented with 0.2% (V/V) BPE, 0.23ng/mL EGF (all supplied with the medium), and CaCl_2_ to a final concentration of 0.13mM. Supplemented hormonal epithelial medium (SHEM) was made up of three parts of low-glucose Dulbecco’s modified epithelial media (DMEM) with pyruvate (Gibco Life Technologies, cat number 21885-025) and one part of Ham’s F12 medium (cat. Number N6658) supplemented with 10% Fetal calf serum (FCS), 1% P/S, 0.4 μg/mL hydrocortisone (cat. Number H2270), 5 μg/mL insulin (cat. Number I9278), 1.4 μg/mL triiodothyronine (cat. Number T5516), 24 μg/mL adenine (cat. Number A9795), and 10 ng/mL EGF (cat. Number E9644) (all provided by Sigma-Aldrich, unless otherwise specified).

For primary corneal cell culture, the scleral layers of the limbal rings were dissected away, and the remaining limbal tissue containing limbal epithelium was cut into (roughly) 1 mm^2^ pieces. For primary conjunctival cell culture, conjunctival tissue was retrieved, the underlying episcleral tissue dissected away, and the remaining tissue containing conjunctival epithelium was cut into 1mm^2^ (roughly) pieces. The resulting tissue pieces were incubated (limbal and conjunctival pieces separately) with 1X trypsin EDTA (Sigma-Aldrich cat. Number T4174) for 20 min in tissue culture incubator. The resulting cell suspension was removed and centrifuged for 5 min at 1000 rpm. The remaining cell pellet was re-suspended in SHEM medium. This trypsinization process was repeated four times using the same tissue pieces. The resulting cell suspensions were then pooled together and added to tissue culture well containing previously mitotically inactivated murine J2-3T3 fibroblasts [by incubation with 10μg/mL mitomycin C (Sigma-Aldrich, cat. Number M4287) for 2 h at 37°C 5% CO_2_]. The co-cultures were maintained at 37°C, 5% CO_2_ in a tissue culture incubator and SHEM medium changed on the day after and then every other day.

### Extracellular matrix exposure

Following culture of cells for 9 days, they were treated with 20 mM NH_4_OH (cat. Number A669-212, Fisher Scientific) in sterile PBS for 5 min (31, 32). The resulting cells (‘de-roofed’ cells) were washed away five times in sterile PBS followed by one wash with double distilled water (ddH_2_O). This resulted in cultureware devoid of cells and coated with ECM proteins.

### Scanning electron microscopy

The samples were processed as described by Parekh et al. (33). Briefly, the samples were washed for 5 min in ddH2O two times followed by the addition of 1% aqueous osmium tetroxide. After washing the samples for three times with dH_2_O for 10 min, the samples were washed with ethanol in increasing order of concentrations (30%, 50%, 70%, 90%, and 100%) for three times at a gap of 10 min each and dried using methanol for 10 min twice. The samples were submerged in hexamethyldisilazane (HMDS) in a small glass jar three times for 3 min each. After drying the samples for 1 h in the hood, the samples were mounted into a labeled stub using a conductive carbon disc and the edges were sealed with a silver paint. Samples were stored in a drying cabinet and coated with 1.5 nm platinum (Cressington sputter coated) before imaging. Zeiss Sigma VP SEM (Oberkochen, Germany) was used for image observation and acquisition.

### Cell seeding on extracellular matrix proteins

**FIGURE 2:**
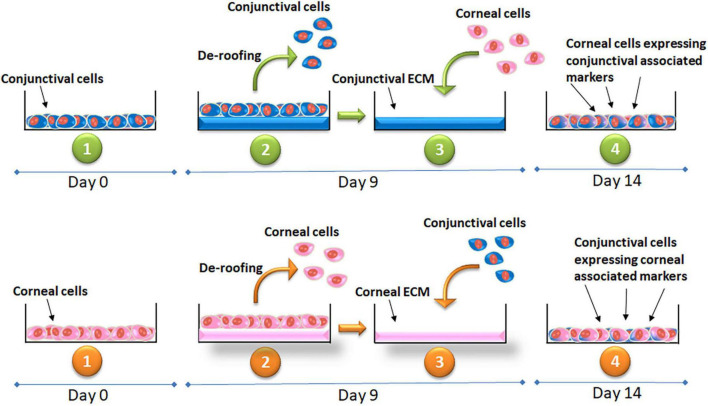
Illustration of the experimental method and timeline showing the cell culture, de-roofing the cells and exposing the extra-cellular matrix of the respective cell lines and culture of corneal epithelial cells on conjunctival ECM and conjunctival cells on corneal ECM. Expression of the markers confirming the cell fate at the end of the experiment.

Fresh cells were seeded on top of exposed/“de-roofed” ECM proteins at a density of 25,000 cells/cm^2^ for 5 days. The tissue culture flasks were maintained at 37°C, 5% CO_2_ in a tissue culture incubator and fed with fresh KSFM (conjunctival or corneal, accordingly) every other day until further experiments (Figure 2). Similarly, primary corneal and conjunctival cells were also cultured on ‘de-roofed’ ECM proteins produced and deposited by primary cells from the same individual whenever feasible.

### RNA extraction, reverse transcription, and real time quantitative polymerase chain reaction

Total RNA was isolated using TRIzol^®^ method. Briefly, the tissue culture well (3.8cm^2^) was incubated with 350μL of TRIzol^®^ reagent (Invitrogen, cat. Number 15596-026) for 5 min at room temperature (RT). The resulting solution was collected, 200μL of chloroform (Sigma Chemicals, cat. Number C2432) was added, the reaction was incubated at RT for 15 min and then centrifuged at 13000rpm for 15 min at 4°C. The colorless aqueous (upper) phase of the centrifuged solution was transferred into a new collection Eppendorf and 500μL of 100% isopropyl alcohol (Sigma-Aldrich, cat. Number I9516) was added. The reaction was then incubated for 10 min at RT and then centrifuged at 13000 rpm for 10 min at 4°C. The supernatant was discarded, and the resulting RNA pellet was washed with 75% (V/V) ethanol (Sigma-Aldrich, cat. Number E7023) in ddH_2_O and centrifuged at 13000 rpm for 5 min at 4°C. The supernatant was discarded, the pellet allowed to air-dry for 15 min at RT and then dissolved in 20 μL of DNAse, RNAse-free water (Ambion, cat. Number AM9937). The quantity and quality of RNA was assessed by analyzing 1.5 μL of the RNA solution using Nanodrop (ND100, Nanodrop Technologies). Subsequent steps for reverse transcription were performed in accordance with the manufacturer’s protocol by Primerdesign using *Precision nanoScript*™ Reverse Transcription kit (all reagents were purchased from Primerdesign). 2 μg of RNA template was used for subsequent analysis. Polymerase chain reaction (PCR) was performed in a LightCycler 480 II (Roche) at 95°C for 2 min for enzyme activation, followed by 45 cycles at 95°C for 15 s, 60°C for 60 s 72°C for 1 s. To assess the purity of the amplicon, the melting curves were performed by continuously acquired fluorescence data until the temperature of 95°C was achieved (at a 0.03°C/s ramp rate). As housekeeping genes gene-to-glyceraldehydes-3-phosphate dehydrogenase (GAPDH) and RPL5 (Ribosomal Protein L5, data not shown) were used for each investigated gene. The fold increase was calculated using ΔΔCt method (34).

### Western blotting

Total protein was isolated using Urea-SDS buffer supplemented with 15% (V/V) β-mercaptoethanol (cat. Number M6250), 50 μM phenylmethanesulfonyl fluoride (cat. Number P7626), and 50 μM N-ethylmaleimide (cat. Number E3876), all provided by Sigma-Aldrich. The protein extracts were then processed for SDS-PAGE. 10% (W/V) acrylamide lower and upper gels were prepared before processing. 5μL of Spectra™ multicolor Broad Range protein ladder (cat. Number 26634 Thermo Scientific) and 20μL of each sample were loaded in different lanes and the gel ran for approximately 2 h at 80V (PowerPac Basic™, BioRad). The Western immunoblotting was run for 2 h at 110V for transfer. The membranes (0.2 μm cat. Number162-0112 BioRad) were thereafter blocked in blocking solution containing 5% (W/V) skimmed milk diluted in 0.05% (V/V) PBSTween-20 (cat. number 9416, Sigma-Aldrich) solution for 30 min and probed with primary antibody diluted in blocking solution overnight at 4°C (Supplementary Table 1). The membranes were then incubated with the secondary antibody diluted in the blocking solution for 1 h at RT (Supplementary Table 2). The two-horseradish peroxidase (HRP) chemicals SuperSignal^®^ West Pico Chemiluminescent substrate (cat. Number 34080, Thermo Scientific) were mixed at ratio of 1:1 and poured over the membrane. The immunoblots were scanned using a Chemidoc™ (chemiDoc™ XRS +, BioRad) and quantified using ImageLab 5.0 Software.

### Flow cytometry

A cell suspension was obtained by trypsinization and then centrifuged (all centrifugation steps were performed for 3 min at 1000 rpm at RT). The supernatant was then removed, and the cell pellet re-suspended in 100 μL of 1X FACS Permeabilizing Solution 2 (cat. Number 347692, BD Biosciences) in ddH_2_O and incubated for 10 min at RT. After washing and centrifugation, the resulting cell pellet was re-suspended in 100μL of primary antibody (Supplementary Tables 3, 4) diluted in PBS and incubated in the dark for 30 min at 4°C and thereafter in 100 μL of appropriate secondary antibody (Supplementary Tables 5, 6) diluted in PBS and incubated in the dark for 30 min at 4°C. After centrifugation, the resulting cell pellet was re-suspended in 500μL of 5% (V/V) FCS in PBS and the data acquire using a BD Accuri C6 flow cytometer (BD Biosciences) and analyzed using BD Accuri C6 software (BD Biosciences).

### Immunocytochemistry on extracellular matrix proteins

Cells were seeded on a glass coverslip (22 × 22mm cat. Number 408/0187/33, Thickness No.1, BDH) over a desired number of days. The wells were washed once in sterile PBS. The cells were then ruptured using 20mM NH4OH for 5 min in sterile PBS. The remaining ECM and basal cell components were washed several times in sterile PBS followed by ddH_2_O. The wells were incubated with ice-cold methanol (cat. Number M/4000/PC17, Fisher Scientific UK) for 10 min at RT (all steps were performed at RT unless otherwise specified), followed by three washing steps with PBS for 5 min. The wells were then blocked using 2% goat serum (cat. Number G9023, Sigma- Aldrich) diluted in PBS for 1 h, followed by three washing steps with PBS for 5 min. Primary antibody diluted in PBS was added and incubated overnight at 4°C in the dark (Supplementary Table 7). The following day, the primary antibody was removed from the culture well and the well washed thrice with PBS for 5 min. The well was incubated with conjugated secondary antibody diluted in PBS for 1 h in the dark (Supplementary Table 8). After removal of the secondary antibody and washing in PBS for 5 min, the coverslip was removed from the well and mounted in a slide (cat. Number 631-0102, SuperFrost 1mm thick, VWR International) using VECTASHIELD (cat. Number H-1000, Vector Laboratories). The coverslips were then viewed using an ECLIPSE Ti-E inverted Microscope System (Nikon, USA).

### Liquid chromatography mass spectrometry

Basal cell protein proteolysis and extraction was performed according to the method described earlier (35) with minor modifications. After cell rupturing with 20 mM NH_4_OH the remaining ECM was washed in sterile PBS followed by ddH_2_O. A solution of 50 mM NH_4_HCO_3_ (cat. number 09830, Sigma-Aldrich) in ddH_2_O, pH 8.0, containing: 0.02% (V/V) ProteaseMax surfactant (cat. number V2071, Promega), 0.33% (W/V) Trypsin Gold (Mass Spec grade, cat. number V5280, Promega) was added and the samples digested for 1 h at 37°C in a humid chamber. The resulting solution (containing the peptides) was then transferred into low-binding tubes (Eppendorf LoBind cat. number Z666505, Sigma-Aldrich). The enzymatic reaction was stopped by adding acetic acid (cat. number 320099, Sigma-Aldrich) setting the pH to 3–4. 1% (V/V) of 0.5M 1,4-dithiothreitol (DTT, cat. number D5545, Sigma-Aldrich) was added for 20 min at 56°C. 2.5% (V/V) of 0.55M iodoacetamide (cat. number I149, Sigma-Aldrich) was added for 15 min at RT in the dark. 0.3125% (W/V) of Trypsin Gold and 0.00625% (V/V) ProteaseMax Surfactant were added and the solution heated at 37°C for 3 h for protein digestion. Trifluoroacetic acid (TFA, cat. number 40967, Sigma-Aldrich) was added to a final concentration of 0.5% (V/V). LC-MS analysis was performed using an Ultimate 3000 RSLCnano system (Dionex, Thermo Fisher Scientific) coupled to a hybrid linear ion trap/Orbitrap mass spectrometer (LTQ Orbitrap XL; Thermo Fisher Scientific). Digested samples were sonicated and 1μg of digested proteins was loaded onto a C18 trap column (C18 PepMap, 300 μm i.d. × 5 mm, 5 μm particle size, 100μm pore size; Dionex) and desalted for 3 min at a flow rate of 25 μL/min using 2% acetonitrile containing 0.1M TFA. The trap column was then switched online with the analytical column (PepMap C18, 75 μm i.d. × 500 mm, 3 μm particle, and 100 μm pore size; Dionex), and peptides were eluted in a 180 min gradient at a flow rate of 300 nL/min using 2% acetonitrile with 0.1% formic acid (FA) to 50% acetonitrile containing 0.08% FA. Mass Spectrometry Data was acquired with Xcalibur software, version 2.0.7 (Thermo Fisher Scientific). The mass spectrometer was operated in data-dependent mode and externally calibrated. MS1 survey scan (m/z 400–1200) was set at a resolution of 30 000 in the Orbitrap, followed by ten MS2 scans using CID activation mode in the ion trap. The dynamic exclusion was enabled with the following settings: repeat count, 1; repeat duration, 30 s; exclusion list size, 500; and exclusion duration, 40 s. The activation time was 30 ms, with an isolation width of 2 Da for ITMS; the normalized activation energy was 32%, and the activation (q) was 0.25.

Proteome Discoverer (PD) version 2.1.0.81 (Thermo-Scientific) was used to perform the database search against the human sequences in the UniProt Swiss-Prot protein database (version January 2016 with 20 151 entries) for the Mass Spectrometry raw data files. The search engines SEQUEST-HT and Mascot (version 2.4.0) were utilized in PD. The search parameters used were as follows: 20ppm tolerance for precursor ion masses, 0.6Da for fragment ion masses analyzed by ion trap. A total of two missed cleavages were permitted for fully tryptic peptides. Carbamidomethylation of cysteines (+ 57.0215 Da) was set as a fixed modification, and variable modifications of methionine oxidation (+ 15.9949 Da) and N-terminal acetylation (+ 42.0106 Da) were allowed. The false discovery rate (FDR) was determined by using a target–decoy search strategy using Percolator in PD2.1. The sequence database contains each sequence in both forward and reverse orientations, enabling FDR estimation. The FDR was set to 0.01 at both the peptide and the protein levels. The LC-MS and data analysis was performed at Dublin City University.

### PathScan^®^ intracellular signaling array kit

PathScan^®^ Intracellular Signaling Array Kit (Chemiluminescent Readout, cat. Number 7323, Cell Signaling Technology) was used for this purpose. All steps were done in accordance to the manufacturer’s protocol at RT. Briefly, cells were cultured for 3 days in different substrates and then washed with ice-cold PBS. Whole protein lysates were prepared using ice-cold 1X Cell Lysis buffer supplemented with a cocktail of protease and phosphatase inhibitors (complete Tablets Mini and PhosSTOP EASYpack, Roche). The Array Blocking Buffer was added and incubated for 15 min on an orbital shaker. The protein lysate was added and incubated for 2 h. After washing, the Detection Antibody Cocktail was added and incubated for 1 h on an orbital shaker. HRP-linked streptavidin was added and incubated for 30 min. LumiGLO^®^/Peroxidase reagent was added and images captured immediately after, using a Chemidoc (chemiDoc™ XRS+, BioRad) and quantified using ImageLab 5.0 Software.

### Statistical analysis

Kruskal-Wallis test followed by a Dunn’s multiple comparison test (unless otherwise specified) were used to determine statistically significant differences (GraphPad Prism 5, *p < 0.05, **p < 0.01, ***p < 0.001). Data is expressed as median ± 5–95% percentile (unless otherwise specified). All experiments were repeated thrice using three technical replicates.

## Results

### Human corneal extracellular matrix proteins drive expression of corneal epithelial and epithelial stem cell markers by human conjunctival epithelial cells as determined by PCR

**FIGURE 3:**
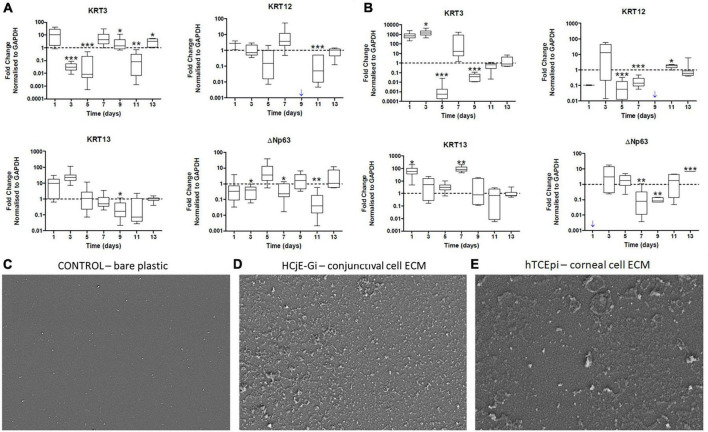
**(A)** The expression of epithelial cell markers by Human conjunctival epithelial cell line (HCjE-Gi) cells when cultured on Human corneal epithelial cell line (hTCEpi) ECM proteins compared to HCjE-Gi ECM deposited over time and **(B)** the expression of epithelial cell markers by hTCEpi cells when cultured on HCjE-Gi ECM proteins compared to hTCEpi ECM deposited over time as assessed by Real Time qPCR. Scanning electron microscopy (SEM) images of **(C)** control (tissue culture flask) without cells or extracellular matrix (ECM). **(D)** SEM image showing the deposited ECM of conjunctival cells (HCjE-Gi) and **(E)** corneal epithelial cells (hTCEpi) at day 9 of the culture. Data is represented as median ± 5-95 percentile, *n* ≥ 9, Mann-Whitney Test, Bonferroni corrected p-value, *p < 0.05, **p < 0.01, ***p < 0.001. Dashed line represents the basal expression of the markers of Interests when cells are cultured on their own ECM proteins. Blue arrow represents expression not detected. GAPDH: glyceraldehyde 3-phosphate dehydrogenase, KRT: keratin, ECM: extracellular matrix.

Cultured hTCEpi cells on tissue culture plates (T) were ‘de-roofed’ at day 9 and HCjE-Gi cells were cultured on the deposited ECM proteins for 14 days. HCjE-Gi cells were cultured on its own ECM protein as a control. The expression of KRT3 was upregulated (1.4-fold increase, *p* < 0.05) and KRT13 was downregulated (6.2-fold decrease) only at day 9. KRT12 expression was not detected when HCjE-Gi cells were cultured on their own ECM proteins at day 9 (blue arrow) (Figure 3A).

### Human conjunctival extracellular matrix proteins drive expression of conjunctival epithelial and epithelial stem cell markers by human corneal epithelial cells as determined by PCR

When hTCEpi cells were cultured on HCjE-Gi cell derived ECM, KRT13 expression by hTCEpi cells was upregulated (87-fold increase, *p* < 0.01) at day 7. Day 9 appeared to be the only time-point where KRT3 expression was downregulated (25-fold decrease) and KRT12 expression was not detected (Figure 3B).

### Deposition of extracellular matrix as assessed by scanning electron microscopy

Scanning electron microscopy of control culture flasks (plastic with no cells) did not show any deposition (Figure 3C) of ECM. However, HCjE-Gi (Figure 3D) and hTCEpi (Figure 3E) derived ECM was observed at day 9 using scanning electron microscopy.

### Human corneal epithelial derived extracellular matrix proteins drive expression of corneal epithelial and stem cell markers by human conjunctival epithelial cell line cells and vice versa

**FIGURE 4:**
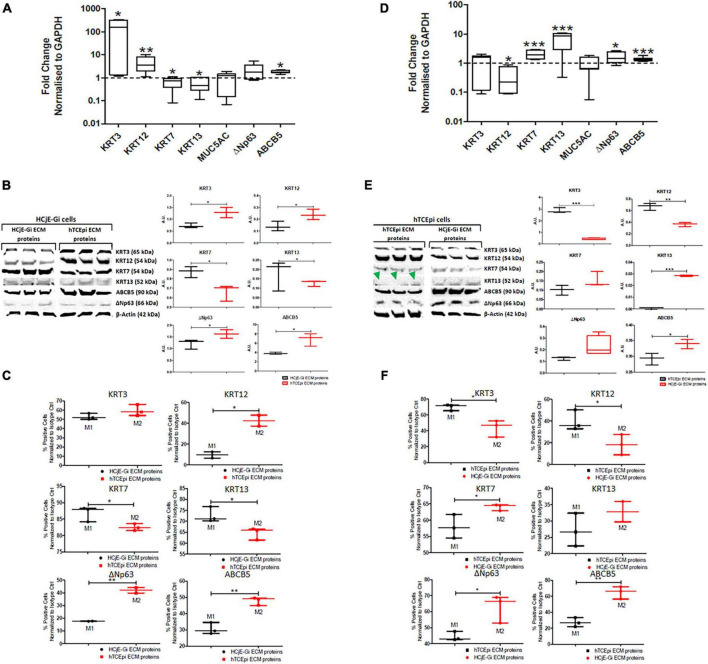
The expression of epithelial cell markers by HCjE-Gi cells when cultured on top of hTCEpi ECM proteins compared with HCjE-Gi ECM **(A)** as assessed by Real Time qPCR. Data normalized to GAPDH levels, **(B)** as assessed by Western blot representative of three independent experiments and densitometry quantification of each protein, normalized against the expression of β-actin and, **(C)** as assessed by flow cytometry. The percentage of positive events normalized against isotype control is shown (Mx). Similarly, the expression of epithelial cell markers by hTCEpi cells when cultured on top of HCjE-Gi ECM proteins compared with hTCEpi ECM **(D)** as assessed by Real Time qPCR. Data normalized to GAPDH levels, **(E)** as assessed by Western blot representative of three independent experiments and the densitometry quantification of each protein, normalized against the expression of β-actin and, **(F)** as assessed by flow cytometry. The percentage of positive events normalized against isotype control is shown (Mx). Green arrowheads show no KRT13 expression when hTCEpi cells were seeded in hTCEpi ECM proteins. Dashed line represents the basal expression of the markers of Interests when cells are cultured on their own ECM proteins. Data is represented as median ± 5-95 percentile, *n* ≥ 6 [for **(A)**] and *n* ≥ 3 [for **(B,C,E,F)**], Mann-Whitney Test, *p < 0.05, **p < 0.01, ***p < 0.001. GAPDH: glyceraldehyde 3-phosphate dehydrogenase, KRT: keratin, MUC: mucin, ABCB5: ATP-binding cassette sub-family B member 5, ECM: extracellular matrix, Ctrl: control.

Both the cell types were cultured for 9 days to obtain the ECM and the respective cells were cultured for 5 days on the respective ECM. HCjE-Gi (conjunctival) cells cultured on hTCEpi (corneal) ECM proteins expressed significantly higher levels of KRT3 (161-fold increase, *p* < 0.05), KRT12 (3.6-fold increase, *p* < 0.01), ABCB5 (1.8-fold increase, *p* < 0.05), and significantly lower levels of KRT7 and KRT13 (respectively, 1.4 and 2.1-fold decrease, *p* < 0.05) by RT-qPCR (Figure 4A). Western blot was consistent with the RT-qPCR data, which showed a significantly higher expression of KRT3, KRT12, ABCB5 and ΔNp63 (*p* < 0.05) accompanied by a significantly lower expression of KRT7 and KRT13 (*p* < 0.05) (Figure 4B). Consistent with this, flow cytometry showed a significantly higher percentage of KRT12-positive cells (30%, *p* < 0.05), ΔNp63- and ABCB5-positive cells (nearly 25% for each marker, *p* < 0.01) accompanied by a significantly lower number of KRT7-positive and KRT13-positive cells (10% for each protein, *p* < 0.05) (Figure 4C). Similarly, hTCEpi cells cultured on top of HCjE-Gi ECM proteins showed significantly lower levels of KRT12 transcript expression (4.0-fold decrease, *p* < 0.05) and significantly higher levels of KRT7 and KRT13 (1.9- and 8.4-fold increase, respectively, *p* < 0.01) and ΔNp63 and ABCB5 expression (1.4-fold increase, *p* < 0.05 and 1.4- fold increase, *p* < 0.001, respectively) (Figure 4D). Western blot showed significantly lower values of KRT3 and KRT12 protein expression (*p* < 0.001 and *p* < 0.01, respectively) accompanied by significantly higher levels of KRT13 (*p* < 0.001) and ABCB5 (*p* < 0.05). The expression of KRT13 was not appreciated (Figure 4E). Using flow cytometry, a significantly lower percentage of KRT3-positive cells (nearly 30%, *p* < 0.05) and of KRT12-positive cells (20%, *p* < 0.05) and significantly higher number of KRT7-positive cells (10%, *p* < 0.05), ΔNp63-positive cells (25%, *p* < 0.05) and ABCB5-positive cells (nearly 40%, *p* < 0.01) was observed following flow cytometry (Figure 4F).

### Primary corneal extracellular matrix proteins drive higher expression of corneal and stem cell epithelial markers by primary conjunctival cells and vice versa

Consistent with the cell line experiments above, significantly higher values of KRT3 expression by RT-qPCR were observed from the conjunctival epithelial cells of all donors (*p* ≤ 0.05), although at lower levels when compared to the fold increase observed in HCjE-Gi cells cultured on top of hTCEpi ECM proteins. The expression of KRT12 in two donors was significantly higher (*p* < 0.01). Significantly lower levels of KRT7 expression (*p* ≤ 0.01) and of KRT13 expression (*p* ≤ 0.05) were shown. The expression of ΔNp63 was significantly higher (*p* ≤ 0.05), and the expression of ABCB5 was only significantly higher in donor REB16R (*p* < 0.01) (Supplementary Figure 1A). The total protein quantification results showed higher expression of KRT3 and KRT12. Lower values of KRT7 and KRT13 expression were also observed. Corneal ECM proteins drove a higher expression of ΔNp63 and ABCB5 (Supplementary Figure 1B). These results are consistent with transcript abundance data. Conversely, by seeding primary human corneal ECs on the HCjE-Gi ECM proteins three outcomes arose. Firstly, significantly lower levels of KRT3 (*p* ≤ 0.01) and KRT12 was observed (*p* ≤ 0.01). Secondly, significantly higher values of KRT7 expression in two donors (*p* < 0.01) and KRT13 expression values (*p* ≤ 0.05) were shown. Thirdly, the expression of ΔNp63 was only significantly higher in one donor (*p* < 0.001), this was accompanied by significantly higher values in the expression of ABCB5 (p ≤ 0.05). Moreover, no ABCB5 expression was detected in two donors when primary corneal cells were seeded on their own ECM proteins (Supplementary Figure 1C). The total protein assessment results showed lower expression levels of KRT3 and KRT12. The expression of KRT7 and KRT13 was higher when cells were cultured on conjunctival ECM proteins. The expression of ΔNp63 and ABCB5 was higher when primary corneal cells were cultured on conjunctival ECM proteins (Supplementary Figure 1D). These results are consistent with the transcript abundance data. As *in vitro* expansion is limited from the donor tissues, only one sample from each donor was used for western blotting. Hence, no statistical analysis could therefore be performed.

### Extracellular matrix composition by mass spectrometry analysis

**FIGURE 5:**
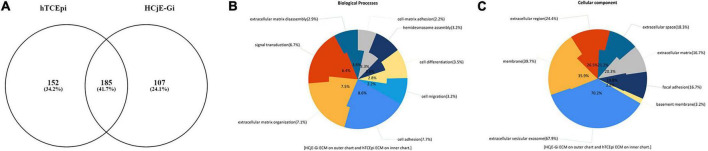
Venn diagram of unique and shared ECM proteome as assessed by mass spectrometry. **(A)** Total of 444 entries were detected: 107 were only found on HCjE-Gi cells-produced ECM, 152 on hTCEpi cells-produced ECM, and 185 detected in both ECM preparations. **(B)** Biological processes associated with the proteins detected on HCjE-Gi and hTCEpi ECM preparations. Inner and outer chart show the percentages relatively to hTCEpi ECM proteins and HCjEGi ECM proteins, respectively. **(C)** Cellular components associated with the proteins detected on HCjE-Gi and hTCEpi ECM preparations. Inner and outer chart show the percentages relatively to hTCEpi ECM proteins and HCjEGi ECM proteins, respectively. Data analyzed using FunRich version 3.0 with Gene Ontology database.

In total 444 different entries were detected: 107 were shown to be found only on HCjE-Gi-produced ECM, 152 exclusively on hTCEpi ECM, and 185 found in both matrices (Figure 5A). From the 107 ECM proteins and growth factors produced by HCjE-Gi and hTCEpi cells, collagen (COL)XVIIα1, LAMβ1, LAMβ2, and LAMα5 would be ideal for further investigation as they have been shown to be present on corneal and conjunctival basement membranes (36). The soluble factors produced by cells and trapped within the ECM proteins meshwork provide chemical cues, additional to those provided by the ECM proteins, for the differential keratin expression needs further investigation. In addition, majority of the ECM proteins seem to be mainly involved in biological processes of cell adhesion, ECM organization and signal transduction (Figure 5B). The main cellular components associated with the proteins detected in both ECM proteins are shown (Figure 5C). Interestingly, presence of a large number of proteins associated with exosomes was observed.

### mRNA expression levels for extracellular matrix proteins show to be different in HCjE-Gi and hTCEpi cells – Real time quantitative PCR and the percentage of positive events for extracellular matrix proteins is shown to be different in HCjE-Gi and hTCEpi cells by flow cytometry

**FIGURE 6:**
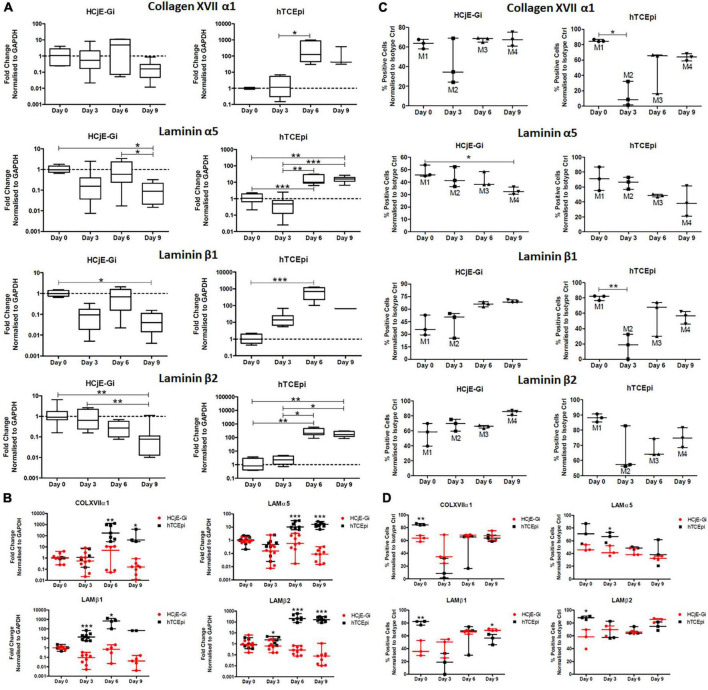
**(A)** The expression of collagen XVII α1, laminin α5, laminin β1 and laminin β2 by HCjE-Gi and hTCEpi cells over 9 days as assessed by Real Time qPCR. **(B)** The expression of transcripts produced by HCjE-Gi and hTCEpi cells over 9 days as assessed by Real Time qPCR. **(C)** The expression of collagen XVII α1, laminin α5, laminin β1 and laminin β2 by HCjE-Gi and hTCEpi cells over 9 days as assessed by flow cytometry. The percentage of positive events normalized against isotype control is shown (Mx). **(D)** The expression of ECM proteins produced by HCjE-Gi and hTCEpi cells over 9 days as assessed by flow cytometry. The percentage of positive events normalized against isotype control. **(A)** Data is represented as median ± 5-95 percentile, *n* ≥ 6, Kruskal-Wallis test followed by a Dunn’s Multiple Comparison test. Dashed line represents the basal expression of the gene of interest at day 0. GAPDH: glyceraldehyde 3-phosphate dehydrogenase. **(B)** Data represented as median ± range, *n* ≥ 3, Mann Whitney test. **(C)** Data is represented as median ± range, *n* ≥ 3, Kruskal-Wallis test followed by a Dunn’s Multiple Comparison test (Data is represented as median ± range, *n* = 3, Mann-Whitney test). **(D)** Data is represented as median ± range, *n* = 3, Mann-Whitney test. *p < 0.05, **p < 0.01, ***p < 0.001. COL: collagen, LAM: Laminin, Ctrl: control, A.U.: arbitrary units, GAPDH: glyceraldehyde 3-phosphate dehydrogenase, ECM: extracellular matrix.

The expression of collagen XVII α1 by HCjE-Gi cells, although not detected on mass spectrometry, was detected by Real Time qPCR since early time points. However, its abundance levels did not vary significantly through time. On the other hand, an increase in its transcript expression levels was observed from day 3 to day 6 on hTCEpi cells (126-fold increase, *p* < 0.05). No significant differences in laminin α5 transcript abundance levels by HCjE-Gi cells were detected until day 9 when it dropped significantly (*p* < 0.05). In contrast, a continuous increase in its expression levels by hTCEpi cells was appreciated from days 0 to 3 until day 6-9 (*p* < 0.01). Laminin β1 trimers with laminin α5 and laminin γ1 to form laminin-511 and consequently their pattern of expression is expected to be similar. Consistent with this, a decrease in its expression level was observed from days 0 to 9 by the HCjE-Gi cells (25-fold decrease, *p* < 0.05). Simultaneously, a continuous increase in its expression levels by hTCEpi cells was observed from day 0 until day 6 when it peaks (698-fold increase, *p* < 0.001). laminin β2 also trimers with laminin α5 and laminin γ1 to form yet a different laminin isoform (laminin-521). A continuous decrease in its expression levels was observed for the HCjE-Gi cells throughout the course of the experiment (*p* < 0.01). Regarding its expression by hTCEpi cells, a peak was observed at day 6 (206-fold increase, *p* < 0.05) when it plateaus (Figure 6A). When the fold change values of the two cell lines are plotted together, additional and complimentary trends are observed. These include higher collagen XVII α1, laminin α5 and laminin β2 transcript levels expressed by hTCEpi cells at day 6 and 9 (*p* < 0.05), and higher laminin β1 and laminin β2 transcript levels expressed by hTCEpi cells at day 3 (*p* < 0.05) (Figure 6B). Protein expression levels in terms of positive events were assessed by flow cytometry to detect both, membrane and cytosolic epitopes. Consistent with Real Time qPCR results, no appreciable changes in the percentage of collagen XVII α1-positive events can be seen on HCjE-Gi cell preparations over time. Regarding its expression by hTCEpi cells, significant lower levels of the percentage of collagen XVII α1-positive events were shown from days 0 to 3 (decrease of 70%, *p* < 0.01), followed by a steady increase until day 9 (not significant). The expression of laminin α5 by HCjE-Gi cells was consistent with Real Time qPCR results, significant lower levels in the percentage of positive events were observed from days 0 to 9 (*p* < 0.05, corresponding to a decrease of 15% in laminin α5 positive events). A decrease in its expression levels was also shown on hTCEpi cells although non-significant throughout the course of the experiment. The abundance of laminin β1-positive events in HCjE-Gi cell preparations were shown not to significantly vary over the time course analyzed. Concerning its expression by hTCEpi cells a significant decrease in the percentage of laminin β1-positive events from days 0 to 3 (*p* < 0.01, reduction of 60% in positive events) was observed. The levels of laminin β2-positive events by HCjE-Gi cells were shown to increase over time (although not statistically different between time-points). Regarding its expression by hTCEpi cells, a decrease in the levels of laminin β2-positive events at day 3 was followed by a constant increase in their expression up to day 9 (not significant) (Figure 6C). A significant higher percentage of COLXVIIα1-positive events at day 0 was detected on hTCEpi cells when fold change values are plotted together (*p* < 0.01, which corresponds to a difference in nearly 20% of COLXVIIα1-positive events between the two cell lines), a significant difference at day 3 on percentage of LAMα5-positive events (*p* < 0.05) equivalent to a difference in nearly 25% more LAMα5-positive events detected on hTCEpi cell preparations. A higher percentage of LAMβ1-positive events at day 0 detected on hTCEpi cell preparations (difference in nearly 40% more LAMβ1-positive events detected in hTCEpi cells, *p* < 0.05), and a difference at day 0 on the percentage of LAMβ2-positive events (*p* < 0.05, corresponding to 30% more LAMβ2-positive events being detected on hTCEpi cell preparations) (Figure 6D).

### Extracellular matrix characterization by immunocytochemistry showed different characteristics at different time points

**FIGURE 7:**
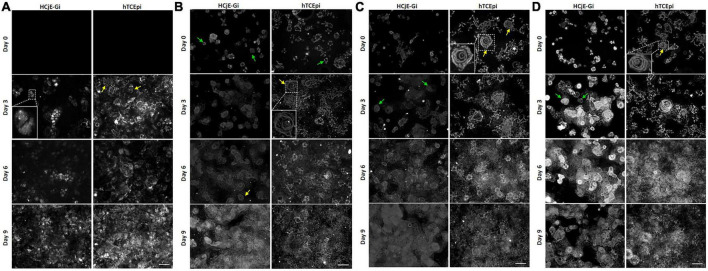
The deposition of **(A)** collagen XVII α1, **(B)** laminin α5, **(C)** laminin β1 and **(D)** laminin β2 by HCjE-Gi and hTCEpi cells over 9 days as assessed by immunocytochemistry. Cells plated onto a glass coverslip were de-roofed and analyzed by immunofluorescence at the showed time-points. **(A)** Box shows the zoomed typical collagen XVII α1 punctated structure. Yellow arrows show a rosette shape resulting from the interaction of collagen XVII α1 with matrix laminins. **(B)** Zoomed box and yellow arrows show a typical laminin rosette structure. Green arrows show an incomplete laminin rosette structure. **(C,D)** Zoomed box and arrows show a typical laminin rosette structure. Scale bar: 50 m.

Absence of COLXVIIα1 at day 0 on both HCjE-Gi and hTCEpi ECM was noted. Its production appears to increase with time in both matrices, showing a punctate pattern (zoomed image). Additionally, on hTCEpi ECM, the pattern soon appears to bear a resemblance to a rosette shape (typical shape of laminin chains with which this protein interacts to form HDs) (Figure 7A). The deposition of LAMα5 increased over time with incomplete rosette shapes observed at day 0 (green arrows) and cloudy appearances at day 9 in both ECMs. The production of this laminin chain appeared to be higher by hTCEpi cells, consistent with the flow cytometry data. The cloudy appearance is result of the high production and deposition of this protein. At intermediate time points, clear rosette shapes were observed (Figure 7B). LAMβ1 (Figure 7C) and LAMβ2 (Figure 7D) chains showed complete rosette shapes at day 3 on HCjE-Gi ECM contrasting with the earlier appearance of these structures on hTCEpi ECM at day 0. Consistent with the flow cytometry data, its deposition appeared to be more accentuated at earlier time points by hTCEpi cells when compared to HCjE-Gi cells.

### General laminin screening

#### HCjE-Gi and hTCEpi cells show different epithelial marker expression when cultured on different human recombinant laminins

HCjE-Gi cells cultured on LAM-111 and LAM-211 showed higher levels of KRT3 transcript levels when compared to those on TCPS (*p* < 0.001); additionally, KRT3 transcript expression showed to be higher in cells cultured on LAM-211 when compared to LAM-121, -221, -411, and -521 (*p* ≤ 0.05). HCjE-Gi cells cultured on LAM-121 showed higher levels of KRT12 expression when compared to those on TCPS. Expression of KRT7 and KRT13 transcripts and their levels appeared to be higher when HCjE-Gi cells were cultured on LAM-111, -211, and -421 than those cultured on LAM-121, -221, -411, -521, and TCPS (*p* ≤ 0.05). No significant differences were appreciated on ΔNp63 levels between the various conditions; although the expression of ABCB5 was shown to be higher on LAM-421 when compared to the other coated proteins (*p* ≤ 0.05). Noteworthy is the absence of ΔNp63 transcript expression by HCjE-Gi cells cultured on LAM-121 and LAM-221 (blue arrows) (Supplementary Figure 2A). hTCEpi cells cultured on LAM-111, -211, -521 showed higher levels of KRT3 expression than those cultured on LAM-411, and those cultured on LAM-511 expressed higher levels of KRT12 than those cultured on LAM-411 (*p* < 0.05). hTCEpi cells seeded on LAM-121, -221, and -411 expressed lower levels of KRT7 than those cultured on LAM-211, -421, and TCPS (*p* ≤ 0.05). hTCEpi cells cultured on LAM-411 expressed lower levels of KRT13 than those cultured on LAM-421, -511, -521, and TCPS (*p* ≤ 0.05). Cells cultured on LAM-411 showed lower expression of ΔNp63 than those on LAM-421 and LAM-511; and lower levels of ABCB5 than those cultured on TCPS. Noteworthy was the absence of KRT12, ΔNp63, and ABCB5 transcript expression by hTCEpi cells when cultured on LAM-221 (blue arrows), ΔNp63 and ABCB5 when in LAM-521 (red arrows) (Supplementary Figure 2B). In general, it appears that cells cultured on LAM-511 did not retain fully differentiated characteristics as seen by the higher expression of putative SC markers, due to its distribution throughout the surface of human eye and its role in maintaining the overlying cells in an undifferentiated state, this isoform was chosen to further investigate its role on modulating EC differentiation.

#### HCjE-Gi and hTCEpi cells respond differently to different concentrations of LAM-511

HCjE-Gi cells were cultured on surfaces functionalized with different concentrations of LAM-511. HCjE-Gi cells cultured on surfaces coated with 10 μg/mL showed higher levels of expression of all keratin transcripts when compared to those seeded on TCPS and on surfaces functionalized with lower LAM-511 concentrations (*p* ≤ 0.05, with some exceptions). KRT12 was not expressed when HCjE-Gi cells were cultured on 2.5 μg/mL coated cultureware (blue arrow). HCjE-Gi cells cultured on 2.5 μg/mL showed higher expression of ΔNp63 (10-fold increase, *p* ≤ 0.05), and 10μg/mL led to higher levels of ABCB5 expression when compared to cells cultured on surfaces coated with 1 μg/mL and 5 μg/mL of LAM-511 (8.8-fold increase, *p* ≤ 0.01), (Supplementary Figure 2C). Similarly, hTCEpi cells cultured on 5μg/mL coated surfaces showed changes in keratin expression profile. Significantly higher levels of KRT3, KRT12, and KRT13 expression on 5μg/mL coated surfaces were observed when compared to cells seeded on others LAM-511 concentrations. hTCEpi cells cultured on lower concentrations of LAM-511 (2.5–5 μg/mL) appeared to express higher levels of ΔNp63 and ABCBC5 when compared to those cultured on other LAM-511 concentrations (Supplementary Figure 2D).

#### HCjE-Gi and hTCEpi cells’ response to LAM-511 functionalized substrates are time dependent

HCjE-Gi cells cultured on LAM-511 for 3 days showed higher expression of KRT3 and ΔNp63 than those kept in culture for 5 days (24- and 2-fold increase, respectively, *p* < 0.001). No KRT12 transcript expression was detected (Supplementary Figure 2E). hTCEpi cells cultured on LAM-511 for 3 days showed higher expression levels of KRT3, KRT7, and ABCBB5 than those left in culture for 5 days (5.1-, 6.4-, and 2.3-fold increase, respectively, *p* ≤ 0.05). Additionally, the transcript abundance of KRT12, KRT7, and KRT13 by hTCEPi cells (12-, 6.4-, and 3.8-fold increase, respectively, *p* ≤ 0.01) was also higher for cells kept in culture for 3 days when compared to those maintained in culture for 1 day only, (Supplementary Figure 2F).

### The protein residues’ phosphorylation and cleavage levels are reduced when cells are cultured on surfaces coated with extracellular matrix proteins

**FIGURE 8:**
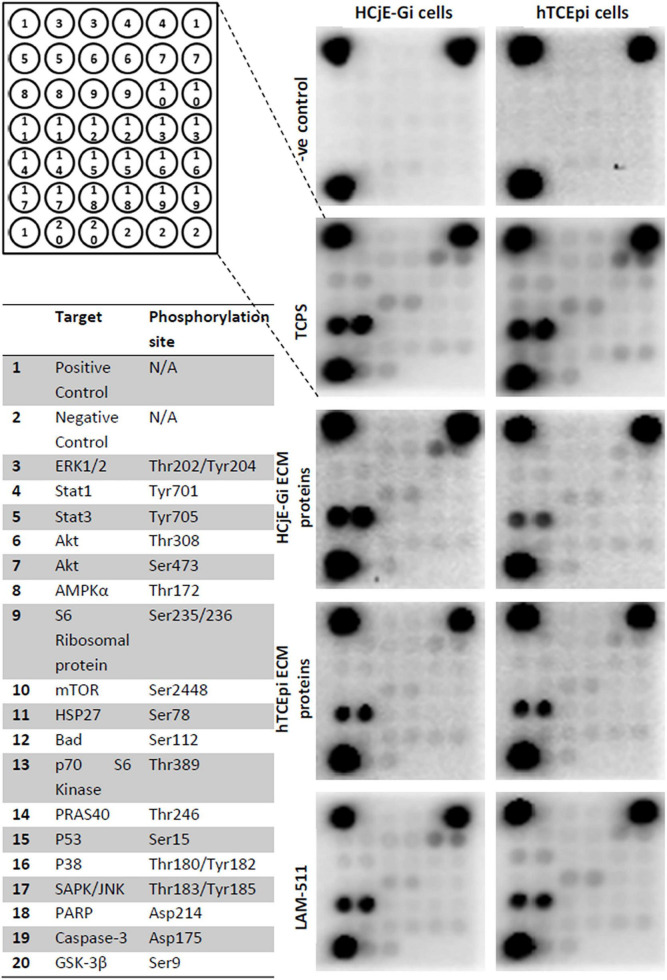
PathScan^®^ intracellular signaling array showing target map, chemiluminescent readout and immunoblotting.

**FIGURE 9:**
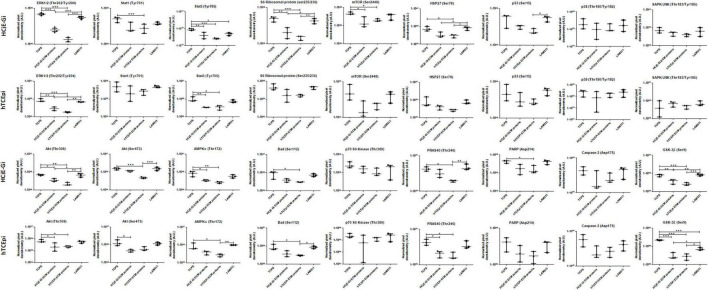
Densitometry quantification of PathScan^®^ intracellular signaling array of HCjE-Gi and hTCEpi cells when cultured in different matrices. Data is represented as median ± 5–95 percentile, *n* ≥ 3, One-way ANOVA followed by Tukey’s Multiple Comparison Test, *p < 0.05, **p < 0.01, ***p < 0.001. TCPS: tissue culture polystyrene, A.U.: arbitrary units, ECM: extracellular matrix, LAM: laminin.

A significant decrease in phosphorylation or cleavage levels of nearly all proteins was observed when HCjE-Gi cells were seeded in pre-coated cultureware, regardless of the ECM proteins preparation. This trend was not observed in the levels of p70 S6 kinase, p53, p38, SAP/JNK, and caspase-3 residue phosphorylation. Very similar results were seen for the hTCEpi cells. Additionally, no changes in STAT1, S6 ribosomal protein, mTOR and PARP residue phosphorylation or cleavage levels were shown when hTCEpi cells were cultured on pre-coated cultureware, (Figures 8, 9).

## Discussion

Real Time qPCR, Western blot, and flow cytometry data showed consistent trends and results. Taken together, the three data sets showed that HCjE-Gi cells cultured on hTCEpi ECM proteins showed higher expression levels of corneal epithelial cell (EC) and stem cell (SC) markers (likely to indicate the emergence of early epithelial progenitor cells) and lower expression levels of conjunctival EC markers when compared to those obtained from HCjE-Gi cells cultured on HCjE-Gi ECM proteins. These data sets suggest that the HCjE-Gi cells, a conjunctival cell line, start to show corneal epithelial-like characteristics, losing its conjunctival epithelial phenotype in a process that involves an intermediate step of cell de-differentiation, an approach suggested by Kragl et al. in other organisms (37).

HCjE-Gi cells cultured on HCjE-Gi ECM proteins also expressed the ‘specific’ corneal epithelium-associated markers, although at lower levels when compared to those cultured on hTCEpi ECM proteins. Two different reasons may help to explain these results. Firstly, the presence of ectopic clusters of KRT12-positive cells in human conjunctival tissue localized in the vicinity of SCs has been shown (38). Secondly, KRT3 has also been shown to be expressed in bovine conjunctival epithelium *in vitro* but not *in vivo* (23). These observations suggest that KRT3 may be antigenically masked *in vivo* and the detected expression may be a result of the KRT3 epitope “unmasking” in *in vitro* cultures, as seen in cells undergoing mitosis (39). This would imply that KRT3 expression in conjunctiva is *in situ* inhibited by some sort of exogeneous factor and upon culture this exogenous factor is somehow lost. The source of this “negative” factor could be the vast blood vessel system present in the conjunctiva *in vivo*, which is absent in the cornea and in the *in vitro* conditions. However, the possibility that there may be an induction of KRT3 production by HCjE-Gi cells *in vitro* should not be ruled out.

The residual expression of conjunctival markers KRT7 and KRT13 by HCjE-Gi cells cultured on hTCEpi ECM proteins suggested that, although the major differentiating lineage is that of corneal epithelium, it may not be the only one, with other epithelial lineages likely to be formed. Another explanation for this arises from the notion that proliferation and differentiation are not mutually exclusive processes, i.e., the expression of a new gene product is not necessarily linked to functional irreversibility (40). These observations suggest that, whilst some conjunctival cells are differentiating toward the corneal epithelial-like lineage, a pool of conjunctival cells may still be proliferating, exhibiting the conjunctival-like characteristics.

On the opposite equivalent direction, the other three data sets suggest that the hTCEpi cells, a corneal cell line, cultured on HCjE-Gi (conjunctival) ECM proteins showed conjunctival epithelial-like characteristics, losing their corneal epithelial phenotype in a process that involves an intermediate step of cell de-differentiation. These results suggest that cell plasticity can be modulated in both ways when cells are provided with appropriate extracellular cues. Previous experiments have led to the suggestion that, upon injury, cells dedifferentiate not into a complete pluripotent state but rather to restricted progenitor cells (37). Tata et al. have shown the de-differentiation of committed airway luminal secretory cells into stable and functional SCs *in vivo* (41). Garcia-Arraras et al. have also shown a process of dedifferentiation of ECs to mesenchymal phenotypes during intestinal regeneration (42). Similar to those observations, the studies in this manuscript suggest that the process of re-differentiation may be preceded by an intermediate state of dedifferentiation as an increase in expression of putative epithelial SC markers was observed. However, dedifferentiation and/or cell division are not obligatory processes in the conversion from one cell type into another (43).

The results obtained using cell lines as models were further confirmed using primary cells. Very similar trends were found, however, with greater variability, intrinsic to the utilization of human primary (non-immortalized) cells. With the exception of KRT3 and ΔNp63, the expression of all markers by the two cell lines were shown to be close to an average value of the widespread values obtained by primary cells. This suggests that the cell lines used have a similar “average” behavior when compared to the primary cells from the tissues that they were extracted from.

Mass spectrometry data showed that ECM composition is cell-dependent, although some proteins were shown to be present in both matrices. The most abundant proteins found in both matrices, and therefore unlikely to be involved in the process of differentiation into specific lineage, include fibronectin, the three chains that compose of LAM-332, various integrin chains, tenascin, and thrombospondins. Fibronectin (11), LAM-332 (11), and integrin α3β1, α6β1, and α6β4 (44) were all also found by others to be present in the conjunctival and corneal basement membrane (BM). On the other hand, tenascin-C and thrombospondin-1, here found in both matrices, are suggested to be enriched in the limbal and cornea regions, respectively (11). Mass spectrometry results showed COLXVIIα1, LAMα5, and LAMβ1 to be specific for either ECMs, and therefore likely to be involved in the process of cell differentiation. Supplementary Table 9 includes a list of basement membrane components across the ocular surface.

COLXVII is a protein involved in the formation of hemidesmosomes and consequently involved in the adhesion of cells to the underlying substrate (45). Mutations in the COLXVII gene are associated with both generalized atrophic benign and junctional epidermolysis bullosa and recurrent erosions of the corneal epithelium (46, 47). The higher expression levels detected in hTCEpi cells suggest a stronger and more cohesive cell-substrate interaction. These results were expected since cornea is a tighter structure when compared to the conjunctiva (9, 30). Others have, however, localized COLXVIIα1, LAMα5, and LAMβ1 within both the corneal and conjunctival BM (11, 48). LAMβ2, on the other hand, has been mostly localized in the conjunctiva (11, 36, 49) and limbal BM (11). The time specificity and the differences in protein production profiles may trigger different cell responses upon culture on different matrices. Others have suggested that even in similar matrices, the organizational states of the common ECM molecules and/or the way such components interact with each other and/or with other soluble factors may provide tissue specificity (23, 50). The results in this manuscript thus not only highlight the potential interactions between the proteins present in the ECM but also their interactions with growth factors and other soluble factors trapped within the protein meshwork, as these are shown not to be washed away by the “de-roofing” method.

Functionally, laminin isoforms are known to regulate the differentiation of ECs (51). Differences in the laminin composition of the conjunctival and corneal BM provide circumstantial evidence for some external modulation of EC differentiation (52, 53). Consistent with these observations, our experiments showed that different recombinant laminin isoforms have different effects on the expression profiles of various EC markers. HCjE-Gi cells cultured on functionalized substrates that contain the LAMβ2 chain showed higher levels of corneal EC markers KRT3 and KRT12 and lower levels of conjunctival EC markers KRT7 and KRT13, when compared to cells cultured on uncoated substrates. These results are in some way contradictory with the work of Kurpakus and Lin, who showed that conjunctival cells cultured in matrices that lack LAMβ2, loss expression of KRT4 (52), a keratin that dimers with KRT13 to form intermediate filaments. Differences in substrate preparation may explain these differences as they used a purified LAMβ2 chain prepared with pepsin from placental laminin whilst this study used a whole laminin isoform that contains that specific chain. Interestingly, it is in the substrates that contain both LAMβ2 and LAMγ1 (LAM-121, -221, and -521) that HCjE-Gi cells expressed higher values of the corneal markers and lower values of conjunctival markers. Very scarce information is published on the effects of LAMγ1 in the ocular cell differentiation. It is present in both the corneal and conjunctival BM; at higher intensity in the latter (however, these are non-quantitative studies) (11) and its knockout is lethal (54) due to the lack of BM formation (55). The results may suggest that HCjE-Gi cells cultured on LAM-111, -211, and -421 are in a less differentiated state as they express not only the conjunctival-associated markers, KRT7 and KRT13, but also the associated corneal markers KRT3 and KRT12. Similar observations were obtained by Wei et al., who by culturing conjunctival ECs on feeder layers observed the expression of KRT5/14, hyperproliferative keratins KRT6/16, small amounts of KRT3 and KRT12, and KRT8/18 (53). Suggesting that in appropriate culture conditions conjunctival ECs do not retain fully differentiation characteristics (53) and consequently they may either be in a hyperproliferative or in a dedifferentiation state.

Conversely, hTCEpi cells cultured on LAM-111, and LAM-511 expressed higher levels of corneal markers and lower levels of conjunctival markers. These two laminin isoforms, together with LAM-332, are found to be most abundant in the corneal BM (56, 57). These observations suggested that the recapitulation of the corneal environment is correlated with the differentiation of corneal ECs, as shown by others using different systems (52). Moreover, when hTCEpi cells were cultured on the conjunctival-associated laminin isoforms [LAM-121, -221, and -521 (11, 58)] the expression levels of the conjunctival markers were not significantly different. Taken together these observations suggest that, despite cell responsiveness to the external environment, individual laminin isoforms may not be sufficient to promote complete cell differentiation into specific lineages.

Nishiuchi et al. have suggested that LAM-511 and LAM-521 are the preferred ligand for all the laminin-binding integrins, on other hand LAM-411 was the poorest ligand, showing the lowest integrin binding affinity (59). These observations may help to understand the lack in keratin expression in the cells cultured on LAM-411 as the cell integrity and adhesion may have been compromised due to the poor cell-substrate interaction. Additionally, Polisetti et al. have shown that epithelial stem progenitor cells cultured on LAM-411 have the lowest covered area, cell adhesion, BrdU proliferation and amount of Ki67-positive cells (proliferating cells) when compared to cells cultured on other laminin isoforms (60). Using a different corneal EC line, Kurpakus et al. have shown that it is integrin α3 chain (detected by mass spectrometry in both ECM preparations) that plays a major role in mediating cellular adhesion to LAM-111 and placental laminin (58), mainly composed of LAM-511 and LAM-521. It is then reasonable to affirm that integrin specificity for laminin isoforms is cell type dependent.

To have a deeper understanding on the mechanisms regulating the intermediate step of cell dedifferentiation, we analyze the phosphorylation and cleavage levels of several proteins that have been shown to be involved in the process of cell differentiation. Others have used LAM-511 as substrates to enable mouse embryonic SC self-renewal for a period of over 5 months (61) or used fragments of laminin-511, termed laminin E8 fragments, as alternatives to Matrigel^®^ or to other feeder layers for culturing human embryonic SCs (62). For this reason, LAM-511 was here used as a substrate to attempt to dedifferentiate the cultured cells and the expression of SC markers compared to the highest timepoint observed in our experiments. Upon extracellular stimulation, the MAPK/ERK signaling module is activated by dual phosphorylation of the Thr202 and Tyr204 residues by the dual specificity kinases MEK1 and MEK2, which lead to EC differentiation (63, 64). Additionally, growth of undifferentiated ECs is enhanced by culture with PD098059, a MEK inhibitor (65), and MEK pathway inhibition promotes murine epithelial SC self-renewal (65). Suggesting that MEK may be involved in controlling epithelial SC differentiation. Studies on PC12 cells have shown that inhibition of ERK activity results in enhanced STA3-mediated transcription (66), suggesting that the antagonism between STAT3 and ERK signaling could account for the effect of PD098059 in epithelial SCs. In accordance with these observations, HCjE-Gi and hTCEpi cells when cultured on coated substrates showed lower levels of ERK1/2, STAT1, and STAT3 phosphorylation, suggesting that these cells lost their differentiated state, gaining undifferentiated characteristics as seen by the high levels of keratin, either corneal and conjunctival, and putative SC markers expression. AKT and mTOR regulation are intricately linked, with AKT functioning upstream of mTORC1 and mTORC2 regulating AKT activation. mTORC2 phosphorylates the AKT serine residues Ser473 and Ser450. Phosphorylation of both serine residues stimulates phosphorylation at Thr308 residue by PDK1 (3-phosphoinositide-dependent protein kinase-1) that leads to full AKT activation, which in turn phosphorylate (activity inhibition) GSK-3β, PRAS40, BAD and S6 ribosomal protein (67, 68), consistent with these observations are the similar trends followed by the phosphorylation profiles of those five proteins. AKT can also activate mTORC1 by phosphorylating PRAS40, thereby relieving the PRAS40-mediated inhibition of mTORC1 (69). S6 ribosomal protein, found downstream p70 S6 kinase reflects mTOR pathway activation and predicts cell cycle progression. The activation (phosphorylation) of these proteins has effects on cell differentiation (70–72). Together, and consistent with the other observations (71, 72), these data support the model where low levels of AKT activation (phosphorylation) inhibit cell differentiation, whereas high AKT activation may be necessary for cell differentiation. GSK-3β inhibitors act as mimetics of Wnt stimulation and consequently support expansion of mouse and human epithelial SCs keeping their undifferentiated state (73). Wnt/β-catenin signaling has also been correlated with an increase in the proliferation of LSCs while maintaining the positive LSCs markers expression (74). The phosphorylation and proteolysis levels of all protein residues point all in one single direction; cells cultured on coated surfaces exhibit undifferentiated characteristics – high levels of different keratins and putative SC marker expression. These include low levels of AMPk, HSP27, and PARP phosphorylation (75–78). When the three different coating methods are compared, a striking trend is observed; “de-roofed” culture systems showed lower levels of protein residue phosphorylation and cleavage when compared to LAM-511 coating substrates. These observations suggest that cells cultured on ECM proteins, resultant from the “de-roofing” method, appeared to be in a less differentiated state than those cultured on LAM-511. This may be explained by the lack of structural and chemical cues on LAM-511 coating method, whereas “de-roofed” cultures systems not only provide morphogenic information resultant from the interaction between all the proteins produced, but also provide a pool of soluble factors produced by cells and trapped within the ECM (as the mass spectrometry results show). These cues have been shown to regulate central cell processes [reviewed in (79)], including cell differentiation. On the other hand, LAM-511, despite lack of structural and chemical cues, has a differentiation inhibitory potential that allows cells to preserve their undifferentiated state (61, 80, 81) as seen by the expression of SC markers (ΔNp63 and ABCB5) however, at lower levels than that from the cells cultured on the “de-roofed” systems. TCPS, here used as control, did not provide any cues, either biochemical or structural, and therefore is the one condition showing higher levels of post-translational protein modifications, mostly indicative of a fully differentiated cell state.

This study is limited to the data obtained using cell lines. Additional studies would be needed to systematically extrapolate the data to primary cultures, as there would be mix of cell populations including the stem cells unlike the cell lines. In addition, separating the stem cell population from the other cells followed by differentiation or phosphorylation profile on the ECM would be important to evaluate beyond 14 days of culture. In addition, ECM proteins are only one component of the environmental cues that would influence the phenotype and function of the cells. Altering the mechanical and topographical parameters to mimic the native tissue would influence the cell response and may capture the true differentiation status of the cells.

In conclusion, the results shown in this manuscript suggest that by providing adequate cues, resultant from the interactions of the different ECM proteins and other growth factors, the plasticity of ECs can be modulated. This approach involves an intermediate step of cell de-differentiation as suggested by the higher expression levels of putative SC markers and lower levels of protein residue phosphorylation and proteolysis. The clinical potential of this approach is appreciable as new functionalized scaffolds could then be manufactured perhaps as bandage contact lenses to induce a more complete conjunctival transdifferentiation toward the corneal epithelial lineage.

## Data availability statement

The original contributions presented in this study are included in the article/Supplementary material, further inquiries can be directed to the corresponding author.

## Author contributions

TR, RS, SK, and SA contributed to conception and design of the study. TR, MP, PM, RS, and KH organized the database. TR, MP, and SA performed the statistical analysis and wrote the first draft of the manuscript. TR, MP, PM, FO’S, RS, SK, KH, and SA wrote sections of the manuscript. All authors contributed to manuscript revision, read, and approved the submitted version.

## Supplementary material

Supplementary Figure 1 The expression of epithelial cell markers by primary conjunctival cells when cultured on top of corneal ECM proteins compared with conjunctival ECM as assessed by **(A)** Real Time qPCR. Data normalized to GAPDH levels (Data is represented as median ± interquartile range, *n* ≥ 10) and **(B)** as assessed by Western blot representative of nine independent experiments and the densitometry quantification of each protein, normalized to the expression of β-actin. The expression of epithelial cell markers by primary corneal cells when cultured on top of conjunctival ECM proteins compared with corneal ECM as assessed by **(C)** Real Time qPCR. Data normalized to GAPDH levels (Data is represented as median ± interquartile range, *n* ≥ 12) and **(D)** as assessed by Western blot representative of nine independent experiments and densitometry quantification of each protein, normalized to the expression of β-actin. Dashed line represents the basal expression of the markers of intersects when cells are cultured on their own ECM proteins. A.U.: arbitrary units, GAPDH: glyceraldehyde 3-phosphate dehydrogenase, KRT: keratin, ABCB5: ATP-binding cassette sub-family B member 5, ECM: Extracellular matrix.

Supplementary Figure 2 The expression of epithelial cell markers by **(A)** HCjE-Gi cells and **(B)** hTCEpi cells when cultured on top of several recombinant laminin isoforms as assessed by Real Time qPCR. The expression of epithelial cell markers by **(C)** HCjE-Gi and **(D)** hTCEpi cells when cultured on top of surfaces coated with various concentrations of human recombinant laminin-511 as assessed by Real Time qPCR. The expression of epithelial cell markers by **(E)** HCjE-Gi cells and **(F)** hTCEpi cells when cultured on top of recombinant laminin-511 over 5 days as assessed by Real Time qPCR. Data is represented as median ± 5-95 percentile, *n* ≥ 6, Kruskal-Wallis test followed by Dunn’s Multiple comparison test, **p* < 0.05, ***p* < 0.01, ****p* < 0.001. Dashed line represents the basal expression of the markers of interests when cells are cultured on TCPS. Arrows represent gene expression not detectable in that laminin isoform/concentration/time point. GAPDH: glyceraldehyde 3-phosphate dehydrogenase, LAM: laminin, KRT: keratin, ABCB5: ATP-binding cassette sub-family B member 5, TCPS: tissue culture polystyrene.
