# Supplementary material for: Specific decellularized extracellular matrix promotes the plasticity of human ocular surface epithelial cells
Source: Front Med (Lausanne). 2022 Nov 15;9:974212. doi: 10.3389/fmed.2022.974212 (PMC9705355; doi:10.3389/fmed.2022.974212)
Supplement: Supplementary file 1 [file Table_1.DOCX]

| **Antibody** | **Clone** | **Host** | **Blocking** | **Manufacturer** | **Dilution** |
| --- | --- | --- | --- | --- | --- |
| KRT1 | Polyclonal | Rabbit | BS | Abcam | 1:1000 |
| KRT3 | AE5 | Mouse | BS | Abcam | 1:1000 |
| KRT7 | RCK105 | Mouse | BS | Santa Cruz | 1:1000 |
| KRT12 | J6 | Rabbit | BS | # | 1:1000 |
| KRT13 | Ks13.1 | Mouse | BS | Santa Cruz | 1:1000 |
| ABCB5 | 5H3C6 | Mouse | BS | Abcam | 1:1000 |
| ΔNp63α | Poly6190 | Rabbit | BS | Biolegend | 1:1000 |
| Β-Actin | mAbcam 8224 | Mouse | BS | Abcam | 1:5000 |

**Supp. Table 1.** List of primary antibodies used for Western Blot studies. Abbreviations used KRT: keratin, BS: blocking solution, ABCB5: ATP-binding cassette sub-family B member 5. # Kind gift from Professor Jonathan Jones (Northwestern University, Chicago, USA).
