# Supplementary material for: Specific decellularized extracellular matrix promotes the plasticity of human ocular surface epithelial cells
Source: Front Med (Lausanne). 2022 Nov 15;9:974212. doi: 10.3389/fmed.2022.974212 (PMC9705355; doi:10.3389/fmed.2022.974212)
Supplement: Supplementary file 3 [file Table_3.DOCX]

| **Antibody** | **Clone** | **Host** | **Isotype** | **Manufacturer** | **Dilution** |
| --- | --- | --- | --- | --- | --- |
| KRT3 | AE5 | Mouse | IgG | Abcam | 1:1000 |
| KRT7 | RCK105 | Mouse | IgG1 | Santa Cruz | 1:500 |
| KRT12 | J6 | Rabbit |  |  | 1:500 |
| KRT13 | Ks13.1 | Mouse | IgG1 | Santa Cruz | 1:500 |
| ABCB5 | 5H3C6 | Mouse | IgG1 | Abcam | 1:1000 |
| ΔNp63 | Poly6190 | Rabbit | IgG | Biolegend | 1:1000 |
| IgG | --- | Mouse | --- | ThermoFisher Scientific | # |
| IgG | --- | Rabbit | --- | ThermoFisher Scientific | # |
| IgG1 | --- | Mouse | --- | ThermoFisher Scientific | # |
| IgG3 | --- | Mouse | --- | ThermoFisher Scientific | # |

**Supp. Table 3.** List of primary antibodies used for flow cytometry studies. Abbreviations used KRT: keratin, ABCB5: ATP-binding cassette sub-family B member 5. # Concentration dependent on primary antibody.
