# Supplementary material for: Specific decellularized extracellular matrix promotes the plasticity of human ocular surface epithelial cells
Source: Front Med (Lausanne). 2022 Nov 15;9:974212. doi: 10.3389/fmed.2022.974212 (PMC9705355; doi:10.3389/fmed.2022.974212)
Supplement: Supplementary file 4 [file Table_4.DOCX]

| **Antibody** | **Clone** | **Source** | **Isotype** | **Manufacturer** | **Dilution** |
| --- | --- | --- | --- | --- | --- |
| COLXVIIα1 | EPR18614 | Rabbit | IgG | Abcam | 1:500 |
| LAMα5 | 2F7 | Mouse | IgG1 | Sigma-Aldrich | 1:500 |
| LAMβ1 | Polyclonal | Rabbit | IgG | ThermoFisher Scientific | 1:500 |
| LAMβ2 | CL2979 | Mouse | IgG2a | Abcam | 1:500 |
| IgG | Isotype control | Rabbit | --- | ThermoFisher Scientific | 0.49ug/uL |
| IgG1 | Isotype control | Mouse | --- | ThermoFisher Scientific | 0.2ug/uL |
| IgG2a | Isotype control | Mouse | --- | ThermoFisher Scientific | 2ug/uL |

**Supp. Table 4.** List of primary antibodies used for flow cytometry studies. Abbreviations used COL: collagen, LAM: laminin.
