# Supplementary material for: Specific decellularized extracellular matrix promotes the plasticity of human ocular surface epithelial cells
Source: Front Med (Lausanne). 2022 Nov 15;9:974212. doi: 10.3389/fmed.2022.974212 (PMC9705355; doi:10.3389/fmed.2022.974212)
Supplement: Supplementary file 6 [file Table_6.DOCX]

| **Antibody** | **Host** | **Reactivity** | **Conjugate** | **Manufacturer** | **Dilution** |
| --- | --- | --- | --- | --- | --- |
| A-11005 | Goat | Mouse | AlexaFlour594 | Life Technologies | 1:400 |
| F7512 | Sheep | Rabbit | FITC | Sigma-Aldrich | 1:400 |

**Supp. Table 6.** List of secondary antibodies used for flow cytometry studies. Abbreviations used FITC: fluorescein isothiocyanate.
