# Supplementary material for: Specific decellularized extracellular matrix promotes the plasticity of human ocular surface epithelial cells
Source: Front Med (Lausanne). 2022 Nov 15;9:974212. doi: 10.3389/fmed.2022.974212 (PMC9705355; doi:10.3389/fmed.2022.974212)
Supplement: Supplementary file 7 [file Table_7.DOCX]

| **Antibody** | **Clone** | **Source** | **Isotype** | **Manufacturer** | **Dilution** |
| --- | --- | --- | --- | --- | --- |
| COLXVIIα1 | EPR18614 | Rabbit | IgG | Abcam | 1:1000 |
| LAMα5 | 2F7 | Mouse | IgG1 | Sigma-Aldrich | 1:1000 |
| LAMβ1 | Polyclonal | Rabbit | IgG | ThermoFisher Scientific | 1:1000 |
| LAMβ2 | CL2979 | Mouse | IgG2a | Abcam | 1:1000 |

**Supp. Table 7.** Table of primary antibodies used for the ICC studies. Abbreviations used COL: collagen, LAM: laminin
