# Supplementary material for: Specific decellularized extracellular matrix promotes the plasticity of human ocular surface epithelial cells
Source: Front Med (Lausanne). 2022 Nov 15;9:974212. doi: 10.3389/fmed.2022.974212 (PMC9705355; doi:10.3389/fmed.2022.974212)
Supplement: Supplementary file 9 [file Table_9.DOCX]

| **BM Component** | **Central cornea** | **Peripheral cornea** | **Anterior limbus** | **Posterior limbus** | **Conjunctiva** |
| --- | --- | --- | --- | --- | --- |
| **Collagens** |  |  |  |  |  |
| **Collagen IV** | + | + | + | + | + |
| **Collagen IV α1 chain** | - | - | + or ++ | + or ++ | + or ++ |
| **Collagen IV α2 chain** | -/+ | -/+ | ++ | ++ | ++ |
| **Collagen IV α3 chain** | + or ++ | + | - | - | - or -/+ |
| **Collagen IV α4 chain** | - or -/+ | - or -/+ | - | - | - or -/+ |
| **Collagen IV α5 chain** | ++ | ++ | ++ | ++ | ++→+→-/+ |
| **Collagen IV α6 chain** | ++ | ++ | ++ | ++ | +→-/+→- |
| **Collagen V** | + | + | - | - | - |
| **Collagen VI** | - | - | - | - | - |
| **Collagen VII** | ++ | ++ | ++ | ++ | ++ |
| **Collagen XVI** | - | + (Sub BM) | ++ (Sub BM) | - | - |
| **Collagen XVII** | + | + | + | + (gaps) | + |
| **Glycoproteins** |  |  |  |  |  |
| **LAM-111** | + | + | + | + | + |
| **LAM-332** | ++ | ++ | ++ | ++ | ++ |
| **LAMα1 chain** | - or -/+ | + | + | + | - |
| **LAMα2 chain** | - | - or -/+ | + | ++ | + |
| **LAMα3 chain** | + | + | + | + | + |
| **LAMα4 chain** | - or -/+ | - or -/+ | - | - | - |
| **LAMα5 chain** | - or -/+ | -/+ or + | + | + | + |
| **LAMβ1 chain** | -/+ or + | -/+ or + | ++ | ++ | + |
| **LAMβ2 chain** | - | - | + | + | + |
| **LAMβ3 chain** | + | + | + | + | + |
| **LAMγ1 chain** | -/+ or + | + | ++ | ++ | ++ |
| **LAMγ2 chain** | ++ | ++ | ++ | ++ | ++ |
| **LAMγ3 chain** | - | - | + | ++ (focal) | + (focal) or - |
| **Fibronectin** | + | + | ++ | ++ | + |
| **Nidogen-1** | + | + | ++ | ++ | ++ |
| **Nidogen-2** | -/+ | + | ++ | ++ | ++ |
| **Clusterin** | + | ++ | - | + | ++ |
| **Thrombospondin-1** | + | + | - | - | - |
| **Thrombospondin-4** | - | + (focal) | + (focal) | + (focal) | + (focal) |
| **Proteoglycans** |  |  |  |  |  |
| **Perlecan** | ++ | ++ | ++ | ++ | ++ |
| **Collagen XV** | + | + | + | + | + |
| **Collagen XVIII** | ++ | ++ | ++ | ++ | ++ |

**Supplementary table 9:** Localization of basement membrane components across the ocular surface. Abbreviations used: – no expression; -/+ weak expression; + moderate expression; ++ strong expression; BM:

basement membrane. LAM: laminin. Adapted from [11].
