# Supplementary figures and images for: Specific decellularized extracellular matrix promotes the plasticity of human ocular surface epithelial cells
Source: Front Med (Lausanne). 2022 Nov 15;9:974212. doi: 10.3389/fmed.2022.974212 (PMC9705355; doi:10.3389/fmed.2022.974212)

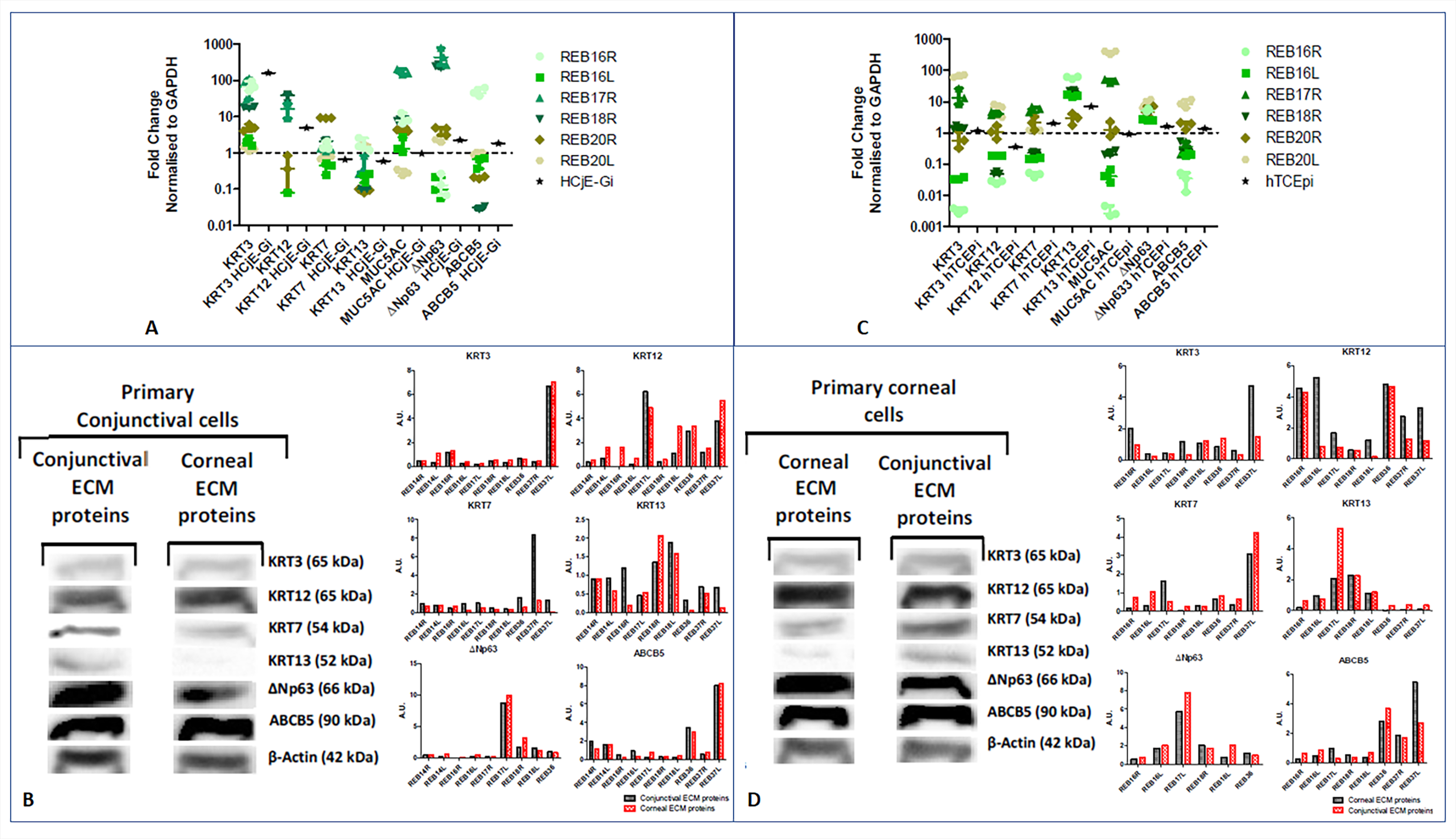

Supplement: Supplementary Figure 1 — The expression of epithelial cell markers by primary conjunctival cells when cultured on top of corneal ECM proteins compared with conjunctival ECM as assessed by (A) Real Time qPCR. Data normalized to GAPDH levels (Data is represented as median ± interquartile range, n ≥ 10) and (B) as assessed by Western blot representative of nine independent experiments and the densitometry quantification of each protein, normalized to the expression of β-actin. The expression of epithelial cell markers by primary corneal cells when cultured on top of conjunctival ECM proteins compared with corneal ECM as assessed by (C) Real Time qPCR. Data normalized to GAPDH levels (Data is represented as median ± interquartile range, n ≥ 12) and (D) as assessed by Western blot representative of nine independent experiments and densitometry quantification of each protein, normalized to the expression of β-actin. Dashed line represents the basal expression of the markers of intersects when cells are cultured on their own ECM proteins. A.U.: arbitrary units, GAPDH: glyceraldehyde 3-phosphate dehydrogenase, KRT: keratin, ABCB5: ATP-binding cassette sub-family B member 5, ECM: Extracellular matrix. [file Image_1.tif]

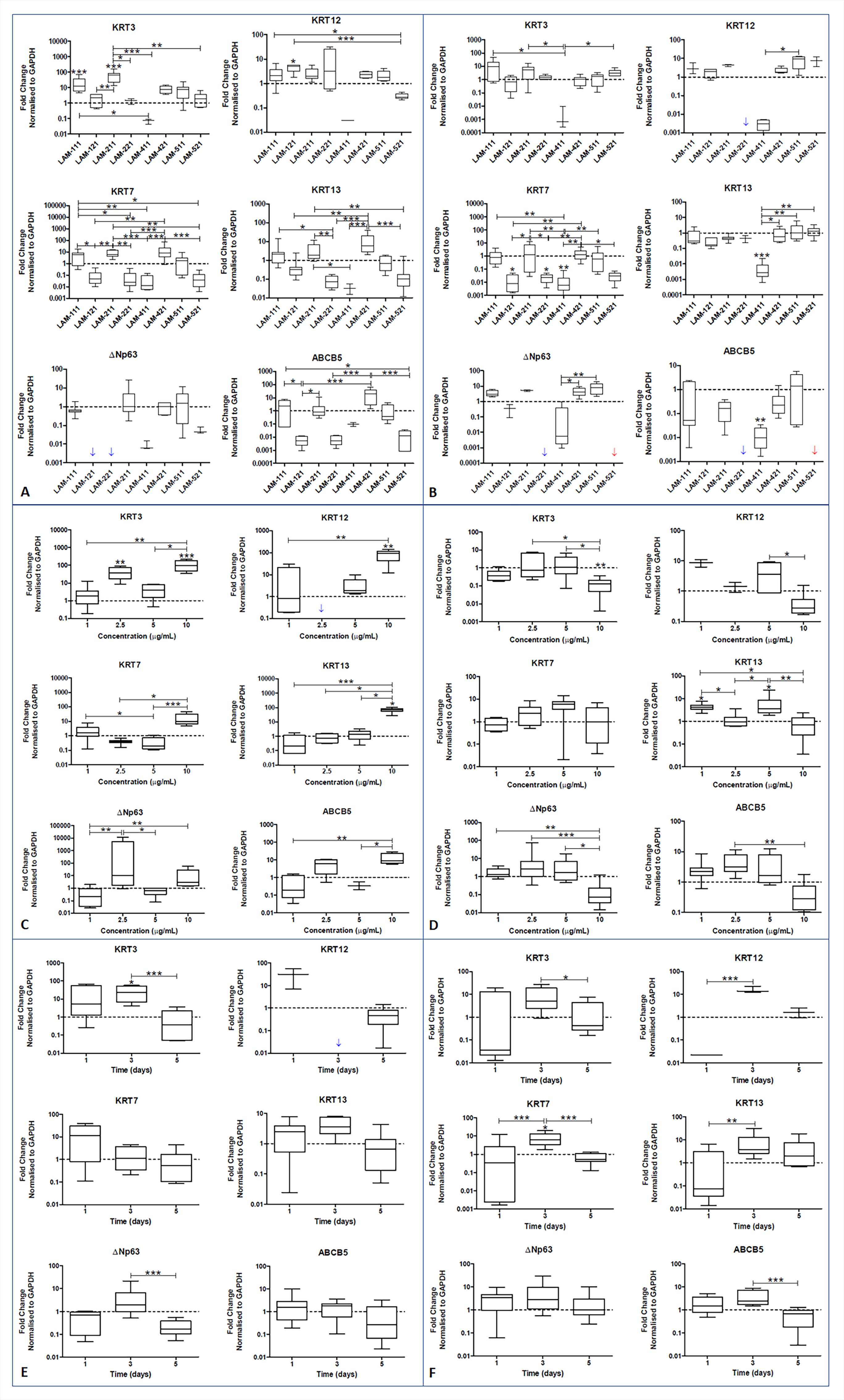

Supplement: Supplementary Figure 2 — The expression of epithelial cell markers by (A) HCjE-Gi cells and (B) hTCEpi cells when cultured on top of several recombinant laminin isoforms as assessed by Real Time qPCR. The expression of epithelial cell markers by (C) HCjE-Gi and (D) hTCEpi cells when cultured on top of surfaces coated with various concentrations of human recombinant laminin-511 as assessed by Real Time qPCR. The expression of epithelial cell markers by (E) HCjE-Gi cells and (F) hTCEpi cells when cultured on top of recombinant laminin-511 over 5 days as assessed by Real Time qPCR. Data is represented as median ± 5-95 percentile, n ≥ 6, Kruskal-Wallis test followed by Dunn’s Multiple comparison test, *p < 0.05, **p < 0.01, ***p < 0.001. Dashed line represents the basal expression of the markers of interests when cells are cultured on TCPS. Arrows represent gene expression not detectable in that laminin isoform/concentration/time point. GAPDH: glyceraldehyde 3-phosphate dehydrogenase, LAM: laminin, KRT: keratin, ABCB5: ATP-binding cassette sub-family B member 5, TCPS: tissue culture polystyrene. [file Image_2.tif]
